# Improving Medicines use in People with Polypharmacy in Primary Care (IMPPP): Protocol for a multicentre cluster randomised trial comparing a complex intervention for medication optimization against usual care

**DOI:** 10.3310/nihropenres.13285.1

**Published:** 2022-11-08

**Authors:** Rupert A. Payne, Peter S. Blair, Barbara Caddick, Carolyn A. Chew-Graham, Tobias Dreischulte, Lorna J. Duncan, Bruce Guthrie, Cindy Mann, Roxanne M. Parslow, Jeff Round, Chris Salisbury, Katrina M. Turner, Nicholas L. Turner, Deborah McCahon

**Affiliations:** 1 Centre for Academic Primary Care, University of Bristol, Bristol, UK; 2 Bristol Trials Centre, University of Bristol, Bristol, UK; 3 School of Medicine, Keele University, Keele, UK; 4 Institute of General Practice and Family Medicine, Medical Center of the Ludwig-Maximilians-University, Munich, Germany; 5 Advanced Care Research Centre, University of Edinburgh, Edinburgh, UK; 6 Institute of Health Economics, Edmonton, Alberta, Canada

**Keywords:** polypharmacy, clinical trial, medicines optimisation, general practice

## Abstract

**Introduction:**

Polypharmacy is increasingly common, and associated with undesirable consequences. Polypharmacy management necessitates balancing therapeutic benefits and risks, and varying clinical and patient priorities. Current guidance for managing polypharmacy is not supported by high quality evidence. The aim of the Improving Medicines use in People with Polypharmacy in Primary Care (IMPPP) trial is to evaluate the effectiveness of an intervention to optimise medication use for patients with polypharmacy in a general practice setting.

**Methods:**

This trial will use a multicentre, open-label, cluster-randomised controlled approach, with two parallel groups. Practices will be randomised to a complex intervention comprising structured medication review (including interprofessional GP/pharmacist treatment planning and patient-facing review) supported by performance feedback, financial incentivisation, clinician training and clinical informatics (intervention), or usual care (control). Patients with polypharmacy and triggering potentially inappropriate prescribing (PIP) indicators will be recruited in each practice using a computerised search of health records. 37 practices will recruit 50 patients, and review them over a 26-week intervention delivery period. The primary outcome is the mean number of PIP indicators triggered per patient at 26 weeks follow-up, determined objectively from coded GP electronic health records. Secondary outcomes will include patient reported outcome measures, and health and care service use. The main intention-to-treat analysis will use linear mixed effects regression to compare number of PIP indicators triggered at 26 weeks post-review between groups, adjusted for baseline (pre-randomisation) values. A nested process evaluation will explore implementation of the intervention in primary care.

**Ethics and dissemination:**

The protocol and associated study materials have been approved by the Wales REC 6, NHS Research Ethics Committee (REC reference 19/WA/0090), host institution and Health Research Authority. Research outputs will be published in peer-reviewed journals and relevant conferences, and additionally disseminated to patients and the public, clinicians, commissioners and policy makers.

**ISRCTN Registration:**

90146150 (28/03/2019)

## Introduction

Polypharmacy is broadly the prescribing of multiple medicines to one individual, and although there is no consensus on how many medicines define ‘multiple’, polypharmacy is increasingly common irrespective of how it is measured ^1^. An ageing population and increasing multimorbidity are key factors driving polypharmacy, compounded by single-condition clinical guidelines recommending more intensive treatment ^2^. Given that the majority of prescribing occurs in primary care, where people with long-term conditions are increasingly managed, polypharmacy presents a particular challenge to general practice.

The use of multiple medications is often considered undesirable by patients ^3^, and can have a number of adverse consequences, including poor medication adherence, adverse drug effects, and increased service use, as well as medication errors, and reduced quality of care ^2, 4– 8^. Importantly, there is a need to differentiate appropriate polypharmacy (where medication use is optimised) from problematic polypharmacy (where medications are used inappropriately or where the intended benefit is not realised) ^9^. Medication optimisation strategies for managing polypharmacy therefore need to balance expected benefits and risks with patient goals and priorities, and should ideally not only demonstrate reductions in potentially inappropriate prescribing but also benefits to patient outcomes.

Recent national guidance on optimising care for polypharmacy has been produced in the UK, but it is not supported by high quality evidence of intervention effectiveness and cost-effectiveness ^10– 13^. Furthermore, there has been significant recent investment in pharmacists working in UK general practice ^14^ to improve effective and safe use of medicines, but again evidence of how best this resource can be utilised in the context of polypharmacy is lacking. A Cochrane review in 2018 identified 32 studies designed to optimise polypharmacy in older patients ^15^. Most of these were multifaceted complex interventions delivered by pharmacists. However, studies were of limited quality and convincing evidence of clinically significant improvements were lacking. Other trials conducted in UK primary care, such as PINCER ^16^ and DQIP ^17^ have demonstrated that prescribing can be improved by review of patients who trigger one or more potentially inappropriate prescribing indicators. However, these interventions focussed on a limited number (<20) of indicators of limited complexity, and it is therefore not known whether the same approach can be applied to optimising polypharmacy.

There is thus a need for general practice-based interventions which can improve the quality and safety of medication use in people experiencing polypharmacy.

## Methods

### Aims

The aims of the IMPPP trial are to evaluate the clinical effectiveness and cost-effectiveness of an intervention to optimise medication use for patients with polypharmacy in a general practice setting, and to examine intervention implementation.

### Outcomes

The primary outcome of the trial is the mean number of potentially inappropriate prescribing (PIP) indicators triggered per patient at 26 weeks after a pre-medication review eligibility check. The list of potentially inappropriate prescribing indicators is provided in the published *Extended data* (Appendix 1) ^18^.

*A priori* secondary outcomes for the trial will also be measured at 26 weeks follow-up, and include quality of life, health service utilisation, and patient medication and safety outcomes (see Table 1 for details).

**Table 1.** Baseline measures and trial outcomes.

Outcome/measure	Instrument	Data source
**Participant socio-demographics**		
Age, gender		GP records
Ethnicity	7-category, based on census classification	Questionnaire ^a^
Socioeconomic deprivation	Area-based (home postcode) using English Index of Multiple Deprivation	Questionnaire
Miscellaneous other	Specific questions on education level, employment status, home circumstances	Questionnaire
**Primary outcome**		
Potentially inappropriate prescribing	>100 prescribing indicators (see *Extended data*, Appendix 1 ^18^)	GP records
**Patient reported outcomes**		
Quality of life	12-Item Short Form Survey (SF-12) ^19^ Visual analogue scale	Questionnaire
Medication adherence (patient- reported)	MARS questionnaire ^20^ Single question 5-point Likert scale	Questionnaire
Medication adherence (prescription refills)	Medication possession ratio ^21^	GP records
Burden of treatment	Multimorbidity Treatment Burden Questionnaire ^22^	Questionnaire
Medication literacy	Specific questions relating to overall understanding of rationale for medicines, plus 14 key therapeutic areas	Questionnaire
**Patient experience of review** (intervention arm only)		
Satisfaction with care	5-point Likert scale	Post-review questionnaire
Confidence in clinician	5-point Likert scale	Post-review questionnaire
Empathy	Selected 4 questions from CARE Measure ^23^	Post-review questionnaire
Shared decision making	9-item Shared Decision Making questionnaire, SDM-Q9 ^24^ 5-point collaboRATE scale ^25^	Post-review questionnaire
**Health and care service utilisation**		
Unplanned hospital admissions	Count of unplanned acute hospital admissions over previous 6 months	HES
Other hospital use	Count of Accident and Emergency (A&E) and outpatient attendances over previous 6 months	HES
Primary care consultation rate	Count of specific consultation types	GP records, Questionnaire
Other service use	Specific questions on frequency and nature of contact with different services (social care, private care, other community care, etc)	Questionnaire
**Patient/medicines safety outcomes**		
Medication-related admissions	Count of medication-related admissions (derived from ICD10 codes T36-T50, X40-44, Y40-Y84 in primary diagnostic position) over previous 6 months	HES
All-cause mortality		GP records ^b^

*Post-review questionnaire delivered to intervention practices only after completion of patient-facing review. All other measures recorded pre-randomisation, immediately post-eligibility check, and at 26 weeks follow-up, with exception of a. recorded pre-randomisation only and b. recorded at 26 weeks follow-up only. HES, Hospital Episode Statistics (national administrative records)*

### Pilot study

A non-randomised pilot study, including three intervention practices and two control practices, has been completed to optimise intervention design and trial processes prior to the main trial. This provided provisional data on rates of potentially inappropriate prescribing and patient participant recruitment and retention. A formative process evaluation has also been carried out including interviews and observations with patients and practice staff. Findings from the pilot work have informed various aspects of the main trial protocol, and are summarised in the relevant sections below.

### Design

**Figure 1.**
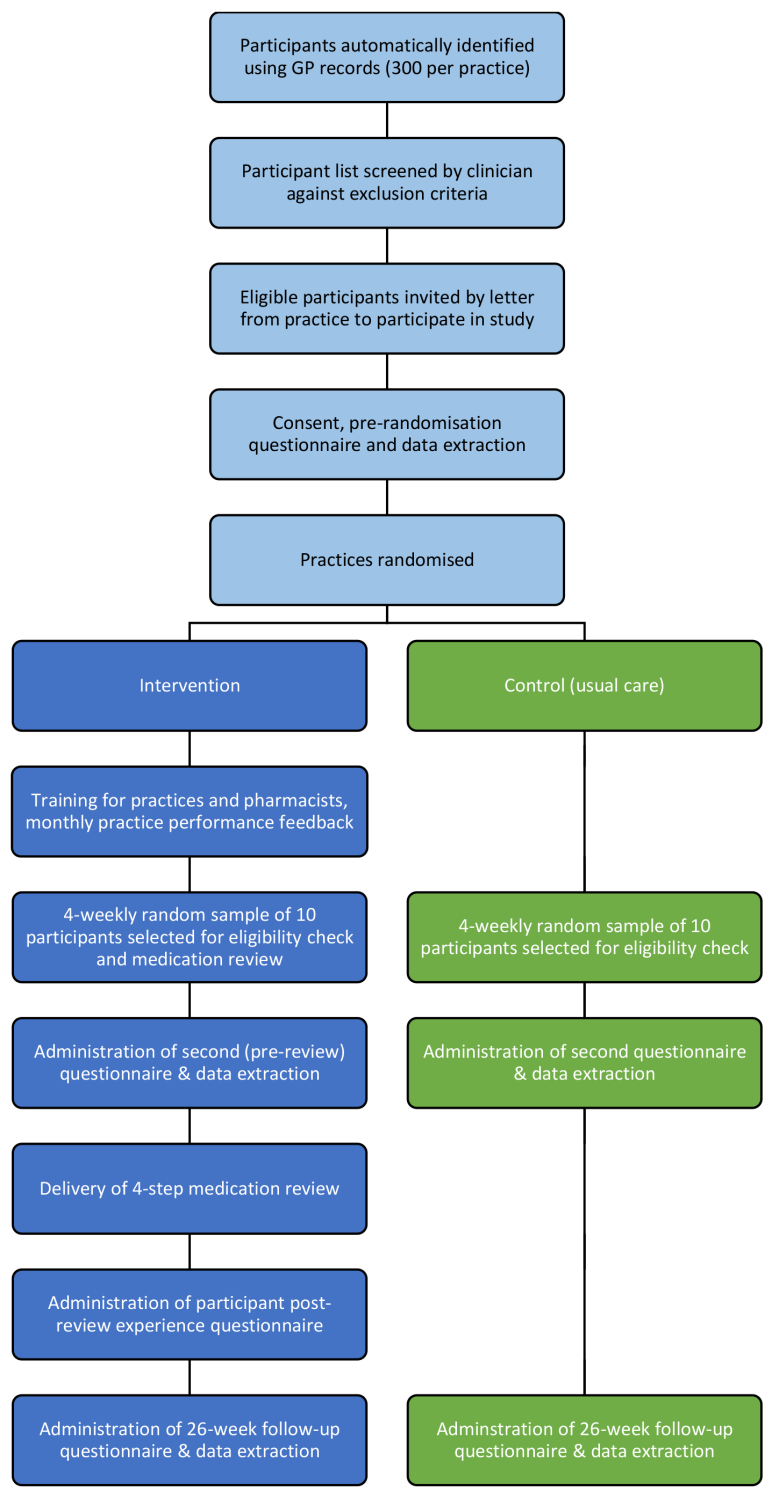
Trial flow diagram.

The trial will be a multicentre, open-label, cluster-randomised controlled trial, with two parallel groups ( Figure 1). Practices will be randomised to usual care (control), or a complex intervention comprising structured medication review involving both a pharmacist and a GP, supported by performance feedback, financial incentivisation, clinician training and clinical informatics (intervention). Patients with polypharmacy and potentially inappropriate prescribing will be recruited in each practice using a computerised search of health records. During the 26-week intervention delivery period, up to 50 patients in each intervention site will receive a medication review. Control practices will continue to provide usual care during this period. Every 4 weeks, over this 26-week period, a random sample of up to 10 consented patient participants in both intervention and control sites will be selected. An administrative eligibility check will be conducted to ensure the patient is still registered at the practice (if not, they will be excluded from the study). All eligible patient participants in intervention sites will then proceed to receive a medication review. Follow-up in both arms will then continue for 26 weeks after the eligibility check. Practices will continue to check eligibility for up to 10 patients every 4 weeks until they have confirmed a maximum of 50 eligible patient participants.

A cost-effectiveness analysis will be conducted. A parallel mixed-methods process evaluation will be undertaken to examine the implementation of the intervention to help explain its success or otherwise, and to inform subsequent implementation in clinical practice.

### Study setting

The trial will be undertaken in UK general practice, in two areas of England (Bristol and surrounding region, and the West Midlands).

### Trial participants

Eligible patient participants will be aged ≥18 years, willing and able to provide written informed consent, with 5 or more medicines listed on “repeat” irrespective of when last issued, and triggering at least one potentially inappropriate prescribing indicator ( *Extended data*, Appendix 1 ^18^). Repeat medications are those recorded in the GP record as being currently available for repeated ordering by a patient without need for further consultation with a clinician; as such, they are generally accepted as representing long-term medication use.

Exclusion criteria will be screened for by a clinician (GP or pharmacist) in the practice, and are: 1) patients receiving end-of-life care; 2) patients judged by the clinician to have chaotic medication use (irrespective of cause); 3) patients for whom the clinician deems contact to be inappropriate (e.g. severe mental health problems, terminal illness, recent bereavement); 4) patients deemed by the clinician unable to complete the study questionnaires or medication review appointment (either themselves or with the help of carers); and 5) patients planning to move general practice within the 26-week follow-up period. We will report numbers of patients excluded in each group. A recent medication review outside the trial setting will not be considered an exclusion criterion, although it will be left to the clinician screening for inclusion in the study to decide if a second review in a short time as part of the study would be clinically appropriate.

### Recruitment of practices

Practice recruitment and randomisation will be undertaken in two geographical areas (Bristol, West Midlands). Practices will be randomised immediately after the initial invitation mail-out to patient participants. This will help minimise the gap between patient consent and practice randomisation. We will recruit 37 practices who will participate in the same pre-randomisation initial set-up period to install the necessary software and provide training in the trial protocol and Good Clinical Practice for safety monitoring and reporting. To reduce risk of contamination, we will avoid recruitment of practices where pharmacists have the potential to work in both intervention and control practices.

### Recruitment of patient participants

Eligible patient participants will be identified by practices after protocol training but before randomisation. All potentially eligible patients meeting the inclusion criteria will be proactively identified using a bespoke informatics tool. This tool will search the GP electronic records to identify those patients who may benefit from the intervention, due to the presence of both multiple medications and potentially inappropriate prescribing. A computer-generated random sample of 300 potential patient participants will be screened by a GP or pharmacist for the exclusion criteria. Based on pilot work, screening resulted in an average exclusion rate of 23%, with subsequent patient acceptance rate of 23% (excluding reminder invitations); screening 300 potential participants is thus expected to provide sufficient recruitment (i.e. 50 participants), particularly when reminder invitations are included. Patients identified as eligible after screening will then be contacted in writing by post, with an invitation letter, participant information leaflet, consent form and baseline questionnaire ( *Extended data*, Appendix 2 ^18^) sent by practices. Those patients willing to participate will be required to return a signed and dated postal consent form; consent will be unwitnessed. If recruitment rates are inadequate after 28 days, a reminder invitation pack will be sent to a random sample of non-responders (number of reminders will be determined by initial response rate). Eligible patients who do not respond to the reminder invitation will not be contacted further regarding the study. If patient participant numbers remain inadequate after reminders, a further case-finding and screening process will be undertaken, with numbers based on the earlier response rates.

### Randomisation and blinding

As soon as initial postal invitations have been sent by a practice, the practice will be randomised. Randomisation will be independently undertaken by computer by the Bristol Clinical Trials Unit. Randomisation will be stratified by region (Bristol, West Midlands). We will not wait for consent forms to be returned before randomising practices; however, we believe the vast majority of patients will remain blind to their practice’s randomisation status at this stage, and it is highly unlikely to influence their consent or baseline questionnaire responses. Potential patient participants sent a reminder will not be informed of the randomisation status of the practice. The study will use as an open-label design, with neither patients nor clinicians blinded to the intervention or study end points. Statistical analysis will be performed blind to practice allocation.

### Description of intervention

**Figure 2.**
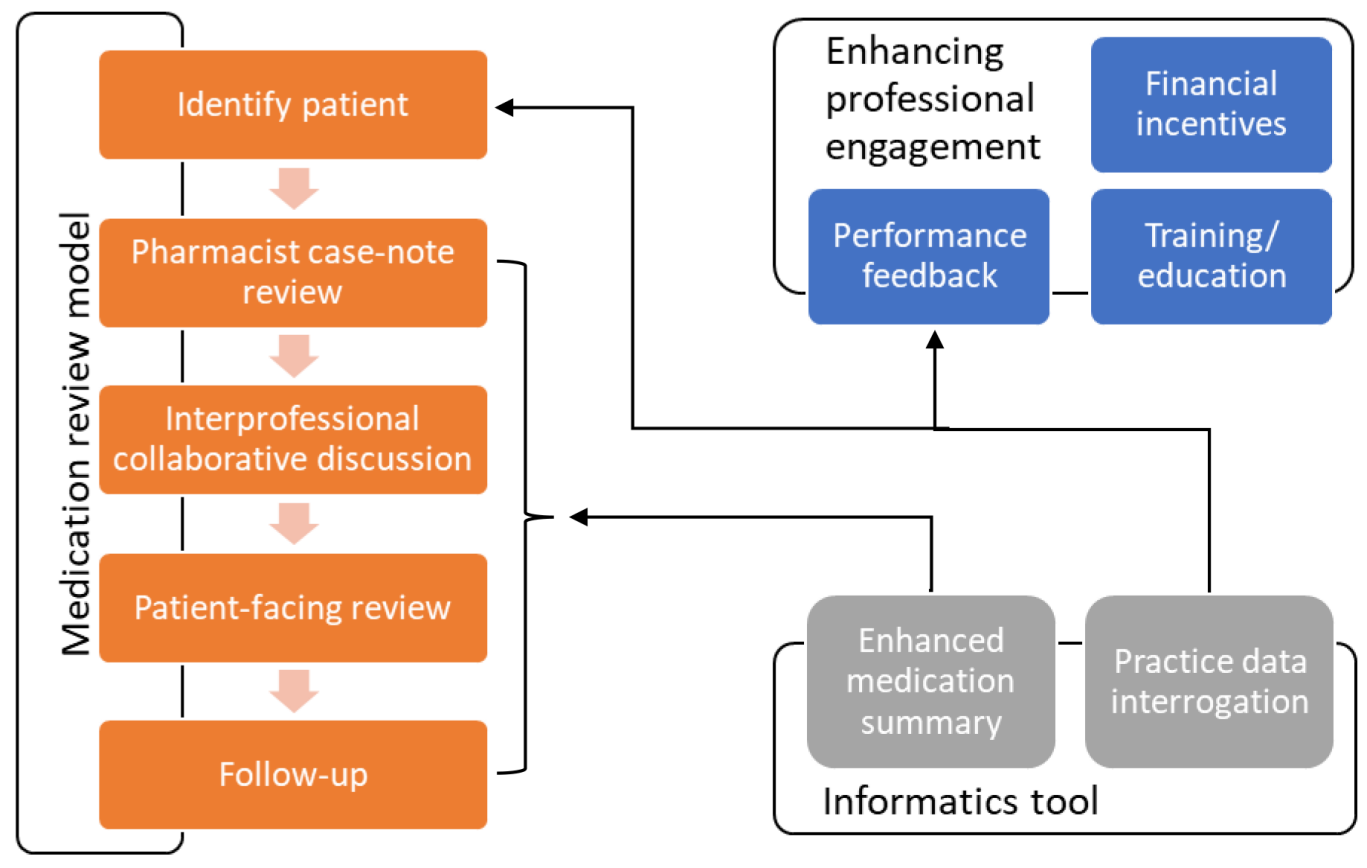
Schematic of IMPPP intervention.

The intervention will take place in general practice, involving GPs and clinical pharmacists working together, drawing on the specific skills of each professional sensitive to the context of each practice. This is a complex intervention and will comprise a model for conducting a polypharmacy medication review (including structured case-finding, pharmacist-GP liaison and collaboration, and a patient-centred approach), and components seeking to enhance professional engagement (clinician training, practice feedback, financial incentives). An informatics tool integrated into GP clinical systems will help support structured case-finding, the medication review itself, and the practice feedback component ( Figure 2). Full details of the intervention are provided in the *Extended data* (Appendix 3) ^18^. Practices will not be explicitly excluded from delivering non-trial medication reviews at other times to participants, if these are clinically indicated, and receipt of a non-trial review will not preclude continued participation in the trial; data on non-trial review activity will be captured for sensitivity analysis.

### Usual care

Practices in the control arm will undertake their usual care for patients with polypharmacy. We anticipate this will comprise routine medication reviews, but no specific management strategy focused on polypharmacy. Control practices will be aware that the participant has consented to a trial of a polypharmacy intervention, but will not be aware of the specific aspect of potentially inappropriate prescribing that has led to the patient’s inclusion. Furthermore, changes to medications which trigger the review process are not mandated as part of the intervention. As such, the trial will not be withholding any clinical intervention targeted at the specific prescribing indicators which would otherwise have triggered a review. Control practices will not have access to the training programme, monthly feedback, computerised enhanced medication summary, or financial incentives. Some of the control practices may have access to a clinical pharmacist, whose role may include medication optimisation.

### Outcome measurements

The primary outcome will be defined as mean number of PIP indicators triggered per participant at 26 weeks following the pre-medication review eligibility check (this check occurs in both trial arms), determined objectively from coded GP electronic health records. For each consented participant, the outcome will be determined at baseline (i.e. pre-randomisation), at the pre-review eligibility check, and at 26 weeks following the pre-review eligibility check.

Secondary outcomes will be determined at the same time points as the primary outcome. Quality of life using the SF-12 instrument, and patient-reported medication adherence and treatment burden, will be ascertained using participant questionnaires. Health and care service use will be determined by a combination of GP records, national hospital utilisation data (NHS Digital Hospital Episode Statistics for Accident and Emergency attendances, in-patient and out-patient care), and questionnaire responses.

Patient demographics will be determined at baseline using participant questionnaires. Additional clinical data (e.g. medication list, recorded clinical conditions) will be extracted approximately 6-monthly using GP electronic health records.

The list of outcome measures, associated data sources, and corresponding measurement time points, is summarised in Table 1. Participants will have the option of responding to the questionnaire either in paper form or electronically. Appendix 2 of the *Extended data* provides a copy of the patient questionnaires ^18^.

Paper questionnaire data will be entered by trained staff. Single data entry will be use, with automated validation checks performed to ensure data quality, plus double-entry checking of a 10% subset of data. The primary prescribing outcome and service usage outcomes will be collected using electronic data extraction.

### Sample size calculation

We consider an average reduction in number of potentially inappropriate prescribing indicators (primary outcome) of 0.5 per participant to be clinically meaningful. Based on data on the distribution of these indicators in pilot work, the standard deviation of the average (mean) indicator count is 2.0. Previous related studies conducted by our research group have also found an intraclass correlation coefficient (ICC) of between 0.0126 (EFIPPS ^26^) and 0.036 (DQIP ^17^). Therefore, to detect a mean decrease in number of indicators triggered per participant of 0.5 in 50 participants per practice, with a power of 90% at the 5% significant level, and assuming an ICC of 0.036, we require a total of 37 practices. Due to the automated manner in which primary outcome data are captured, we expect approximately 100% follow-up; as the follow-up period is only 6 months, we expect few participants to withdraw, move away or die during this time. Even if 20% of cases are lost to follow-up, power will still be 88% at the 5% significance level with the same number of practices. Participant engagement with the medication review process in the pilot study was ≥88%.

### Statistical analysis

The analysis and reporting of this trial will be undertaken in accordance with Consolidated Standards of Reporting Trials (CONSORT) guidelines, as extended to cluster trials. The statistical analyses will follow a pre-defined Statistical Analysis Plan (SAP) agreed prior to the end of the trial. The main primary outcome comparative analyses between randomised arms will be conducted on an intention-to-treat (ITT) basis without imputation of missing data.

The main analysis will use a linear mixed effects regression model to compare number of PIP indicators triggered at 26 weeks (after the pre-medication review eligibility check) between groups as randomised, adjusted for baseline (i.e. pre-randomisation) values of the outcome, area (stratification variable), and elapsed time between baseline and post patient-facing review 26-week follow-up. A random effect for GP practice will be included to account for clustering. The result of the regression model will be presented as an adjusted difference in mean between the intervention and control arms alongside the associated 95% confidence interval and exact p-value for the comparison. If the assumptions of the regression model do not hold then transformations of the data or alternative models (e.g. zero-inflated Poisson or negative binomial regression) will be explored.

Additional sensitivity analyses of the primary outcome will include further adjustment of the main analysis for any prognostic variables strongly related to outcome (identified *a priori* based on clinical expert opinion, e.g. age, number of medications). A further additional analysis will be performed where baseline is taken from the pre-review time-point, with adjustment for pre-randomisation outcome values. This is proposed as there is the potential for a considerable delay between randomisation and review; medication optimisation processes during this time may vary between arms (e.g. usual care may be more likely to take action to improve prescribing, as unlike the intervention arm these practices will be aware that no IMPPP review will be occurring later) resulting in differences in prescribing immediately prior to review which are unrelated to the intervention itself.

The effect of the intervention on the secondary outcomes collected at 6 months post-review follow-up will also be examined using appropriate mixed effects regression models adjusted for baseline values of the outcome being investigated, elapsed time between baseline and 26-week post-review follow-up, area (stratification variable) and including a random effect to account for clustering by practice **.**

The sensitivity of the main analysis to the impact of missing data (where missingness is >5%) will be explored by imputing missing primary outcome data and repeating the primary analysis model using the imputed data. The imputation model will include all variables that are part of the ITT primary analysis, baseline and post-randomisation variables that are associated with missingness, and interim data on the primary outcome collected at 3 months follow-up. It is emphasised that levels of missing data are expected to be low, as the primary outcome is captured using automated processes.

Complier average causal effect (CACE) analysis using a 2-stage-least-squares (2SLS) instrumental variable (IV) approach will be used to investigate the efficacy of the intervention in reducing potentially inappropriate prescribing indicators at 6 months. The CACE methodology compares outcomes for those who “complied” with the intervention with a comparable group of “would be compliers” in the control group. CACE analysis provides an estimate of the efficacy of the intervention for comparison with the ITT estimate of the offer of the intervention, whilst respecting randomisation and avoiding biases inherent to crude per-protocol analyses. In this trial “compliance” will be defined as undergoing the face-to-face medication review. To investigate potential moderators of treatment effect, interaction terms for treatment group by age, and treatment group by multimorbidity will be added (separately) to the primary analysis model.

### Patient and Public Involvement

Patient input informed the design of this study. Two lay representatives will sit on the trial steering committee, and a lay advisory group will provide additional input, including interpretation of process evaluation findings, input into educational practice and patient-facing materials, consideration of ethical and regulatory aspects, identification of non-academic routes for dissemination and contribution to wider public materials.

### Safety reporting

Given the nature of participants in the IMPPP trial (older individuals with polypharmacy and therefore likely with multimorbidity), new diagnoses, hospital admissions and death are to be anticipated. An independent Data Monitoring Committee (DMC) will be established to assess trial safety and efficacy, consisting of three independent academic members including two statisticians and a clinician with relevant interests. Serious adverse events will be monitored, recorded and reported in accordance with the Good Clinical Practice (GCP) guidelines and the Sponsor’s Research Related Adverse Event Reporting Policy. Adverse events may be reported by participant or investigator, and assessment of intensity, relatedness and expectedness will be made by the Principal Investigator at each research site for all serious adverse events. Suspected unexpected serious adverse reactions (SUSARs) will be reported to the coordinating centre within 24 hours, and discussed at each scheduled DMC meeting. SUSARs will be additionally reported to the trial Sponsor within 5 days and the Research Ethics Committee within 24 days. As there is a potential for *ad hoc* adverse events to be more readily identified in the intervention arm, the Principal Investigator at all research sites (irrespective of randomisation status) will undertake an additional proactive review of all participants’ hospital admissions, GP consultations and deaths during the study period and at the end of study follow-up. This will help identify differences in adverse event rates between study arms.

An adverse event will be deemed to have occurred where a change to the prescribed medication is required to counter a change in medication made at the initial intervention (patient-facing review) appointment. An adverse event will be considered expected if a) an adverse drug reaction (ADR) has occurred (i.e. consistent with the WHO definition of ADR, listed in the British National Formulary (BNF) ^27^ as common or very common, and the drug or an interacting drug has been changed as a consequence of the IMPPP intervention), b) worsening of the drug clinical indication has occurred (i.e. where the indication is listed in the BNF, and the drug or an interacting drug has been changed as a consequence of the IMPPP intervention), or c) there is an adverse change in patient behaviour considered by the clinician to be consistent with that expected as a direct consequence of the intervention. Unexpected events will include ADRs considered uncommon, rare or unlisted in the BNF, or an intensity of event greater than that clinically expected, or a worsening of clinical condition despite a clinically appropriate drug change.

### Process evaluation

A mixed-methods process evaluation will be undertaken to examine key trial processes ^28^ (adoption, delivery/fidelity, clinician and patient response, maintenance, and context) giving insight into how the intervention is implemented in order to aid interpretation of the trial results. Analysis of data relating to the key hypothesised mechanisms of action identified in the formative stage will give insight into why the intervention was effective or not.

Quantitative measures will be used to examine delivery of the intervention components by the practice in the intervention arm. This will include assessing clinician experience of the educational outreach component using a post-course participant evaluation questionnaire, degree of practice engagement with the review process (based on practice-level data on completion rates for different elements of the review process), nature of the review process at a patient-level (including which clinician undertakes activity and whether in-person or otherwise), and the number and type of medication changes made. A questionnaire will be sent to all patient participants in the intervention arm within two weeks of completion of the patient-facing medication review to explore patient experience ( Table 1). Additional quantitative measures in both trial arms will examine the trial processes themselves, including practice representativeness, recruitment rate, retention rate, patient completion of baseline and follow-up questionnaires, and surveys to explore practice usual care policies (repeated at the end of the study in the usual care practices), and (in intervention practices only) the nature of pharmacist/GP collaborative interactions.

Qualitative evaluation will also be conducted, with purposeful sampling of a subset of around six to nine intervention practice case studies. Observations will be undertaken of the educational outreach sessions, GP and pharmacist inter-professional collaborative discussions, and patient-facing reviews. After the first five reviews have been undertaken (to allow for familiarisation with processes), semi-structured interviews will also be undertaken over the remaining intervention delivery period. Interviews will be conducted with clinicians, pharmacists and patients/carers in these case-study practices to understand experience of medication review, as well as practice systems, usual clinical care, and contextual issues. Qualitative data will be analysed in parallel with other ongoing data collection, so that emerging issues can be incorporated in future interviews as the study progresses. Data analysis will involve both within-practice and cross-practice analysis, incorporating rich description of implementation in individual case study practices, and cross-case thematic analysis ^29^ of recurring issues relevant to intervention implementation. Particularly for the thematic analysis, NVivo V.11 software (QSR International, RRID:SCR_014802) will be used to facilitate both deductive and inductive coding, allowing the identification of overarching themes. Findings relating to mechanisms of action and to the key components of the intervention, including how they were adopted, delivered, received and maintained, will help to interpret trial results and give an indication of reasons why the intervention worked or otherwise.

### Economic analysis

An economic analysis will be performed using individual participant-level data from the trial. The primary outcome for the economic evaluation will be reported as quality adjusted life years, as derived from SF-12, and converted to SF-6D scores ^30^ using a validated scoring algorithm available online ^31^. The analytical approaches will take the form of cost-effectiveness and cost-utility analyses. Results of the primary economic analyses will be reported as the net-benefit statistic; ICERs and cost-effectiveness acceptability curves will also be reported. The primary analysis will be from the perspective of the NHS and personal social services, with secondary analysis from a societal perspective. We will also estimate cost-effectiveness ratios based on the cost per incremental change in the primary outcome. The cost per unit of change in PIP indicators will also be calculated, using change in the count of potentially inappropriate prescribing indicators at 26-weeks follow-up. The association between change in potentially inappropriate prescribing and change in quality adjusted life years (QALY) during the same period will be reported.

### Ethics and governance

This study will be conducted in accordance with Good Clinical Practice guidelines, UK Policy Framework for Health and Social Care Research, and other relevant legislation (e.g. Data Protection Act 2018). The Sponsor is University of Bristol Research and Enterprise Development (reference 2018–2188). The sponsor and funder will have no role in the study design, writing of papers, or the decision to submit papers (including this protocol) for publication. Monitoring and auditing will be in accordance with the Sponsor’s policy. Protocol review has been undertaken by a NHS Research Ethics Committee (Wales REC 6, reference 19/WA/0090), and the study sponsor has reviewed the study procedures and ensured all indemnity and insurance requirements for the trial were in place prior to the start of recruitment. The current protocol version is 4.0. Protocol amendments will be approved by the REC and Sponsor. Participants will provide written informed consent, including options for participating in the process evaluation, and for sharing anonymous data for subsequent research.

Any medication optimisation strategy targeted at polypharmacy may result in a change (including reductions) in treatment; this is considered ethically acceptable assuming clinical decisions are made in agreement with the patient, are clinically justifiable, and are in the patient’s best interests. These conditions are met in the case of this trial since the study protocol does not pre-specify medication changes, which remain entirely at the discretion of clinicians and their patients.

In addition to the DMC described above, oversight of the trial will be undertaken by an independent Trial Steering Committee, chaired by a senior academic GP, including relevant methodological expertise, and clinical and patient representation, as well as representation from the trial team (RP, CS) as non-independent members.

The trial is registered with the ISRCTN registry (reference number ISRCTN90146150, registration date 28/03/2019).

### Data management and information governance

Data will be collected and retained in accordance with UK data protection legislation. Data storage will conform to the University of Bristol data security policy. Electronic data (including qualitative recordings and transcripts) will be kept on password protected, encrypted servers. Written data will be stored in a secure filing system. Personal identifiable data will be kept separate to clinical and other trial data. The Chief Investigator in conjunction with the Trial Manager and Senior IT manager will manage access rights to data. Once completed, anonymised trial data will be made available for sharing with other researchers once appropriate governance approvals are in place. Essential study documents (e.g. consent forms) and anonymised data will be stored for the period of time specified by local and/or national clinical trial policies.

### Dissemination

Findings from the research will be disseminated to patients and the public (press releases, social media, trial website), health care professionals (workshops and publications through professional bodies), commissioners and policy makers (workshops, policy briefings), and academia (open-access peer-reviewed scientific journal publications, conferences). Authorship of scientific publications will be consistent with the International Committee of Medical Journal Editors Recommendations for the Conduct, Reporting, Editing, and Publication of Scholarly Work in Medical Journals (2018).

### Current trial status

The trial commenced participant recruitment on 24/1/2022, and is estimated to continue recruiting until July 2022. It is anticipated that follow-up of all participants will be completed by July 2023.

## Discussion

Medication review using a patient-centred approach is central to the medication optimisation process recommended by the Royal Pharmaceutical Society (RPS) ^32^, with evidence that medication reviews can improve medication-related problems ^33^. The IMPPP intervention seeks to optimise review delivery through a number of strategies. Firstly, by facilitating identification of the at-risk population, assessing a wider range of potentially inappropriate prescribing than many existing approaches. Secondly, drawing on pharmacists’ expertise in medications and GPs’ skills in relation to multimorbidity management, whilst overcoming recognised barriers to effective interprofessional collaborative working ^34, 35^. Thirdly, by better engaging patients prior to review and facilitating greater patient-centred involvement in discussion and decision making around medication review. Fourthly, by engaging a strategy of social support to facilitate the management of clinical complexity and uncertainty ^36^. Fifthly, through the use of informatics to support a structured review process by ensuring relevant information is collated in an accessible manner to help the reviewer focus attention on key problems.

The IMPPP intervention also includes three components aiming to enhance professional engagement in order to support adoption and effective implementation. Drawing on the COM-B model of behaviour change ^37^, these components help achieve change by improving capability (training for clinicians), opportunity (provision of informatics and pharmacist resources), and motivation (through education, performance feedback, and financial incentivisation). Educational outreach and feedback have been shown to have small but consistent and potentially important effects on prescribing ^38, 39^. Incentives form part of normal contractual arrangements in the UK, and have been incorporated in other successful prescribing safety interventions ^17^.

The intervention design thus helps address a number of challenges with current approaches to effective management of polypharmacy, aligns with current health service systems and processes, and is potentially readily scalable. The study will establish whether and how the IMPPP intervention is effective and cost effective, and will provide valuable insights into optimal implementation. Findings from the IMPPP trial will be generalisable to the UK population, with the potential to achieve change in health service delivery and outcomes, by improving prescribing and quality of life in a substantial proportion of patients.

## Data availability

### Underlying data

No underlying data are associated with this article.

### Extended data

Open Science Framework: Improving Medicines use in People with Polypharmacy in Primary Care (IMPPP). https://doi.org/10.17605/OSF.IO/KMRPW ^18^.

This project contains the following extended data:

- Appendix 1 (List of potentially inappropriate prescribing indicators)
- Appendix 2 (Patient-facing trial documents)
- Appendix 3 (Intervention description)

Data are available under the terms of the Creative Commons Zero "No rights reserved" data waiver (CC0 1.0 Public domain dedication).
