# Supplementary material for: Improving Medicines use in People with Polypharmacy in Primary Care (IMPPP): Protocol for a multicentre cluster randomised trial comparing a complex intervention for medication optimization against usual care
Source: NIHR Open Res. 2022 Nov 8;2:54. [Version 1] doi: 10.3310/nihropenres.13285.1 (PMC10593356; doi:10.3310/nihropenres.13285.1)
Supplement: Peer review history

# Reviewer report: Reviewer response for version 1

**DOI:** 10.3310/nihropenres.14406.r29542

**Published:** 2023-07-14

**Authors:** Concepción Violán

**Affiliations:** 1 Universitat Autonoma de Barcelona, Barcelona, Catalonia, Spain

**General comments:**

 The research protocol answers very well the questions required in open research (NHIR). 

 However, some improvements can be made. The improvements are related to aspects to be considered to enhance the reproducibility of the study and minor concretions in some sections. 

**Introduction:**

 After 1st sentence of the 2nd paragraph of the introduction, “The use of multiple medications is often considered undesirable by patients, and can have a number of adverse consequences, including poor medication adherence, adverse drug effects, and increased service use, as well as medication errors, and reduced quality of care2,4–8”. I would suggest adding some examples of adverse rug effects (Medication-related problems, mortality...).

**Trial Participants:**

 In the 1st paragraph, 2nd sentence “Repeat medications are those recorded in the GP record as being currently available for repeated ordering by a patient without the need for further consultation with a clinician; as such, they are generally accepted as representing long-term medication use”. That includes medications prescribed by another medical specialist (like a cardiologist, dermatologist, etc). Understanding the role or intervention capacity of other physicians is crucial. It will allow us to determine whether the intervention is reproducible or needs to be adapted when implemented in other study populations.

**Description of intervention:**

 The Extended data (Appendix 3) describes financial incentives for practices. There is no mention of incentives for patients. Did patients receive economic incentives? This could potentially influence the results. The Hawthorne effect could also be present.

 It is also mentioned that "Practices will not be explicitly excluded from delivering non-trial medication reviews at other times to participants" and "data on non-trial review activity will be captured for sensitivity analysis." It would be beneficial to specify how these variables/data will be considered, as they can significantly influence the results.

**Usual care:**

 Some of the control practices may have access to a clinical pharmacist, whose role may include medication optimisation. It would be advisable to specify how the presence of a clinical pharmacist, whose role may involve medication optimization, will be considered in the sensitivity analysis.

**Outcome measurements:**

 The final phrase of this section is “The primary prescribing outcome and service usage outcomes will be collected using electronic data extraction.” Is it a system different from the usual electronic record? Is it software designed for the study.

**Economic analysis**

 The authors' comment, "The analytical approaches will take the form of cost-effectiveness and cost-utility analyses," would specify which costs will be considered (direct costs? indirect costs? Infrastructure, services?). Healthcare systems vary greatly, and having this information would be valuable for future studies.

**Discussion**

 It is very well structured in terms of strengths and expected results. However, some limitations should be considered. It is already mentioned that the study only lasts 26 weeks, but it could be mentioned that to address this limitation, once the project results are obtained, polypharmacy will be reviewed in both groups. A follow-up at 12 months could be considered. The problem with these designs is that the intervention is not maintained over time.

**Extended Data, Appendix 1**. 

 It is very well structured regarding strengths and expected outcomes. However, some limitations should be considered. It is already mentioned that the study only lasts for 26 weeks. The possibility of reassessing patients at a later time period should be discussed as a future research direction. The issue with these designs is that the intervention is not sustained over time.

 Finally, I would like to congratulate the authors as I believe this study has been meticulously designed, taking into account all the factors and variables that could potentially lead to confusion in the interpretation of the results. I am looking forward to reading the results soon.

Is the study design appropriate for the research question?

Yes

Is the rationale for, and objectives of, the study clearly described?

Yes

Are sufficient details of the methods provided to allow replication by others?

Yes

Are the datasets clearly presented in a useable and accessible format?

Yes

Reviewer Expertise:

Primary Health Care

I confirm that I have read this submission and believe that I have an appropriate level of expertise to confirm that it is of an acceptable scientific standard.

# Reviewer report: Reviewer response for version 1

**DOI:** 10.3310/nihropenres.14406.r29177

**Published:** 2023-05-16

**Authors:** Barbara Clyne, Aisling Croke

**Affiliations:** 1 Dept. Public Health & Epidemiology, School of Population Health, RCSI University of Medicine and Health Sciences, Dublin, Ireland; 2 General Practice, RCSI University of Medicine and Health Sciences, Dublin, Ireland

This manuscript presents a protocol for a multicentre cluster randomised trial comparing a complex intervention for medication optimization. Overall, it is a well-written and comprehensive paper. Minor clarifications listed below may increase clarity for the readers. 

**Abstract:**

- Under the methods, can you update “37 practices will recruit 50 patients” to “37 practices will recruit a maximum of 50 patients each” for clarity.

**Methods:**

*Eligibility:*

- The authors state “repeat medications” - can you please elaborate on this definition, for example do you intend to count medications with active ingredients only, or will you include diabetic/colostomy/urostomy products as individual medications in the count? Clarification would be important as a diabetic patient may be incorrectly designated as a polypharmacy patient and only be taking two medications alongside needles, test strips and lancets.
- What is the rationale for excluding chaotic medication use. Would a structured medication review not benefit these patients?

*Recruitment: *

- Practices will be randomised immediately after the initial invitation mail-out to patient participants to minimise the gap between patient consent and practice randomisation. While this is logistically sensible, it may introduce selection bias in terms of who GPs will contact when checking eligibility for up to 10 patients every 4 weeks and reminders for non-responders. Can the authors comment on this?

*Analysis: *

- The primary outcome analysis will be intention-to-treat (ITT) without imputation of missing data. A sensitivity of the main analysis to the impact of missing data will be explored by imputing missing primary outcome. If the plan is to impute missing data anyway, what is the rationale for the primary analysis to be ITT without imputation? In the presence of dropout, the validity of a complete-case ITT analysis assumes that there is no selection bias by study dropout. But if those who drop out of the study are "sicker" than those who do not (which may be the case in this trial) then this doesn’t hold true anymore.
- Will the analysis of secondary outcomes also be ITT without imputation? The patient questionnaire packs appear quite detailed. Do authors anticipate missing data due to incomplete questionnaires or non-participation as a result and will it be imputed.
- Consents and questionnaires may not be returned before randomisation, have the authors considered the impact of potential recruitment delays on the trial?
- Are all patient reviews to be conducted in-person or by other means? It is reasonable that patients may be reviewed by video link or telephone, but the protocol seems to indicate that reviews are intended to be conducted in-person. If you could please clarify the following sections:
  - Pg. 6, trial participants; exclusion criteria: “patients deemed by the clinician **unable to complete the study questionnaires or medication review appointment**”
  - Pg. 8, analysis section, final paragraph, line 12: “compliance will be defined as undergoing the **face-to-face medication review**”
  - Pg. 9, process evaluation, quantitative measures paragraph: Nature of the review process at a patient level (including which clinician undertakes the activity and **whether in person or otherwise**).

*Process evaluation:*

- pg. 9, first paragraph; could you please detail what the key hypothesised mechanisms of actions are?

Is the study design appropriate for the research question?

Yes

Is the rationale for, and objectives of, the study clearly described?

Yes

Are sufficient details of the methods provided to allow replication by others?

Yes

Are the datasets clearly presented in a useable and accessible format?

Not applicable

Reviewer Expertise:

Evidence syntheses and systematic reviews, evidence based decision making, the design and conduct of randomised controlled trials, older people and ageing, multimorbidity and appropriate prescribing.

We confirm that we have read this submission and believe that we have an appropriate level of expertise to confirm that it is of an acceptable scientific standard.
